# Relations between task delegation and job satisfaction in general practice: a systematic literature review

**DOI:** 10.1186/s12875-016-0565-1

**Published:** 2016-11-30

**Authors:** Helle Riisgaard, Jørgen Nexøe, Jette V. Le, Jens Søndergaard, Loni Ledderer

**Affiliations:** 1 Research Unit of General Practice, Department of Public Health, University of Southern Denmark, J.B. Winsløws Vej 9A, 5000 Odense C, Denmark; 2 Section of Health Promotion and Health Services, Department of Public Health, Aarhus University, Bartholins Allé 2, building 1260, 225, 8000 Aarhus C, Denmark

**Keywords:** Task delegation, Job satisfaction, General practice, Nurse’s role, Systematic review

## Abstract

**Background:**

It has for years been discussed whether practice staff should be involved in patient care in general practice to a higher extent. The research concerning task delegation within general practice is generally increasing, but the literature focusing on its influence on general practitioners' and their staff’s job satisfaction appears to be sparse even though job satisfaction is acknowledged as an important factor associated with both patient satisfaction and medical quality of care. Therefore, the overall aim of this study was 1) to review the current research on the relation between task delegation and general practitioners' and their staff’s job satisfaction and, additionally, 2) to review the evidence of possible explanations for this relation.

**Methods:**

A systematic literature review. We searched the four databases PubMed, Cinahl, Embase, and Scopus systematically. The immediate relevance of the retrieved articles was evaluated by title and abstract by the first author, and papers that seemed to meet the aim of the review were then fully read by first author and last author independently judging the eligibility of content.

**Results:**

We included four studies in the review. They explored views and attitudes of the staff, encompassing nurses as well as practice managers. Only one of the included studies also explored general practitioners' views and attitudes, hence making it impossible to establish any syntheses on this relation. According to the studies, the staff’s overall attitude towards task delegation was positive and led to increased job satisfaction, probably because task delegation comprised a high degree of work autonomy.

**Conclusions:**

The few studies included in our review suggest that task delegation within general practice may be seen by the staff as an overall positive issue contributing to their job satisfaction, primarily due to perceived autonomy in the work. However, because of the small sample size comprising only qualitative studies, and due to the heterogeneity of these studies, we cannot draw unambiguous conclusions although we point towards tendencies.

## Background

In order to respond to the relative shortage of general practitioners (GPs) in the western world and their continuously increasing workload, it has been discussed for years whether practice staff should be involved in patient care within general practice to a higher extent [1]. Additionally, from various sides, there has been an expectation that delegating tasks is feasible without compromising the quality of care for patients [1, 2]. In many countries, these expectations concerning task delegation have already resulted in changes of the division of tasks in general practice. Task delegation is defined as an intentional transfer of clinical tasks from the GP to another healthcare professional, or another employee with clinical training, within the staff [2].

Quality of care is traditionally perceived as the quality of the medical care provided by physicians and healthcare staff or as satisfaction with the care received by patients [3]. This perspective on quality of care has previously been reviewed regarding task delegation in general practice, and it has been found that the process of care, comprising standards of care, practitioner adherence to clinical guidelines, and practitioner healthcare activity, such as examinations and provision of advice, is as good as or better with nurses than with GPs [4]. The patient perspective has been reviewed as well, concluding that satisfaction with care provided by healthcare staff, primarily nurses, generally seems to be higher than with GP care [4–8].

Another aspect of quality of care is GPs’ and their staff’s job satisfaction which has in literature been recognised as important since it is associated with both patient satisfaction [9, 10] and medical quality of care [10]. Hence, there is evidence that low job satisfaction is associated with suboptimal healthcare delivery provided by GPs [10] as well as adverse events and reduced patient adherence [11]. Moreover, studies have shown that there is an association between low job satisfaction and professional burnout among GPs [12] and between low job satisfaction and a higher turnover of staff [11]. Thus, in order to ensure good quality of care when delegating tasks within general practice, job satisfaction of the GPs and their staff should be taken into consideration as well.

Job satisfaction is often explained as an outcome of the difference between an individual’s expectations, and what the individual actually experiences [13]. It can be defined as the affective orientation one has towards his or her job, either as a global feeling about the job or as a related constellation of attitudes about various aspects or facets of the job [14]. According to this definition, job satisfaction can be defined on different strata, but to understand what job satisfaction is all about, one has to focus on the latter of the two.

Therefore, to examine the relation between task delegation and job satisfaction within general practice, views and attitudes of the GPs and their staff towards this change in the working structure should be the subject of attention. There are several articles exploring views and attitudes towards teamwork in general practice [15–17], primarily in a nurse perspective, but the literature regarding task delegation and its influence on job satisfaction of GPs and their staff appears to be sparse, and to our knowledge it has never been the focus of a review.

Thus, the overall aim of this study is 1) to review the current research on the relation between task delegation and GPs’ and their staff’s job satisfaction and, additionally, 2) to review the evidence of possible explanations for this relation.

## Methods

The review is based on the step-by-step approach described by Harris S. Cooper [18]. This approach has previously been used as the underlying method in a similar review summarizing subjective evidence across highly different studies [19]. Cooper proposes seven steps encompassing formulation of the problem, search of literature, gathering of information from the studies, evaluation of the quality, analysis and integration of outcomes, interpretation of evidence, and finally presentation of the results [18].

### Search of literature

We combined search terms for task delegation with terms for job satisfaction and terms for general practice. The electronic databases, PubMed, Cinahl, Embase, and Scopus, were searched for retrieval of relevant studies. Two experienced librarians assisted in developing the search strategy adjusted for all four databases. For an example of the search strategy as it was developed for PubMed, see Table 1. We searched the databases from their inception until November 2015 which was the month where the searches were conducted. We were interested in studies from international peer-reviewed scientific journals, and therefore we limited our search to English language. We added Scandinavian languages to our search strategy since all of the authors are able to read it and thereby exploring if there were any local studies. Additionally, a Google search was conducted, a hand search was carried out, reference lists of included articles were looked through, and relevant author names were searched to identify more articles.

**Table 1:** Search strategy (PubMed)

Variable	Search terms
Task delegation	(personnel delegation OR task delegation OR delegation, professional OR delegate tasks OR skill mix OR skill-mix OR job substitution OR job division OR job transfer OR nurse's role OR nurse role OR role nurses OR nurse roles OR nurses’ roles OR nurses’ role OR nurse led OR nurse-led OR nurse practitioner OR nurse practitioners OR task shifting OR task division OR division tasks OR task transfer OR role revision OR revision roles) AND
General practice	(general practice OR family physician OR family physicians OR general practitioner OR general practitioners) AND
Job satisfaction	(job satisfaction OR work satisfaction OR provider satisfaction OR professional satisfaction)
Language filter	English, Danish, Norwegian, Swedish

Studies addressing the overall aim, finding evidence of a relation between task delegation and job satisfaction, were eligible for inclusion in the review. The studies had to be conducted within general practice settings, and they could be qualitative as well as quantitative as long as they met the overall aim. Moreover, the studies should concern staff with clinical training substituting or working complementarily to GPs, for instance practice nurses, clinical nurse specialists, nurse practitioners, health care assistants, pharmacists, midwives, medical laboratory technicians, or secretaries with clinical training.

### Gathering of information from the studies and evaluation of the quality

The immediate relevance of the retrieved articles was evaluated by title and abstract by first author HR. Papers that seemed to meet the aim of the review were then fully read by first author, HR, and by author LL independently judging the eligibility of content.

We did not apply a standardised appraisal tool since we valued all potential contributions to the review regardless of study design and methods, and since research shows that a structured approach does not lead to a higher level of agreement among reviewers than an unprompted judgement [20]. Instead we used a narrative approach to identify primary themes and issues of importance. Data extraction was conducted by the authors HR and LL independently. The extracted data in combination with the narratives were then tabulated to provide an overview of the results and to explicate the interpretative process.

### Eligibility

To be eligible for inclusion in the review, the studies had to meet the important initial criteria listed in Table 2.

**Table 2:** Initial criteria for inclusion in the review

Initial criteria
Meet the overall aim of finding evidence of a relation between tasks delegation and job satisfaction of GPs or/and their staff
Conducted within general practice
Focus on healthcare professionals, either GPs or staff with clinical work
Conducted within everyday clinical care and not in relation to an intervention etc.
Published in an international peer-reviewed journal

### Quality assessment

If the content of the study seemed to meet the overall aim, finding evidence of a relation between tasks delegation and job satisfaction of GPs and their staff, the quality was assessed individually according to the principle of degree of “…correspondence between methods and desired inferences” [18], which means that we judged each study on the degree to which the method used was able to answer the research question put forward.

Moreover, we assessed the quality of the eligible studies using the quality criteria listed in Table 3.

**Table 3:** Quality criteria for inclusion in the review

Quality criteria
Coherence between study design and study aim
Coherence between study methods and study aim
Appropriate sample size when using a quantitative approach
Appropriate response rate when using survey data
Process from data collection to reporting of the results described thoroughly
Results presented properly

## Results

**Fig. 1:**
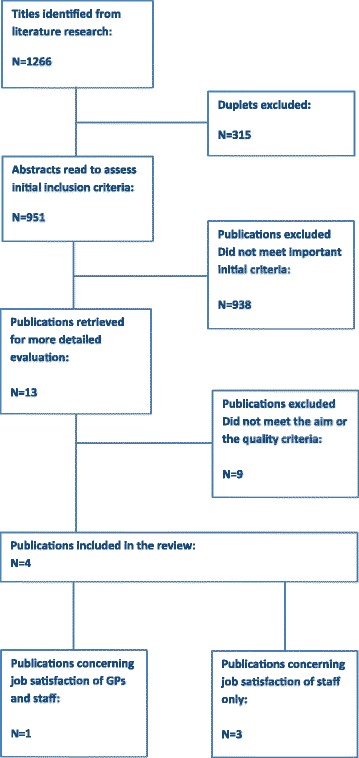
Flow chart

The search revealed 1266 articles. After checking for duplicates, 951 papers were left for evaluation of titles and abstracts. This left 13 articles for reading of full texts. Of these, five papers did not meet the aim [15, 21–24], another two were not conducted within general practice settings [25, 26], and one was not about task delegation [27]. The remaining five articles were read thoroughly to appraise them according to the quality criteria. Only one of them did not meet the quality criteria due to a very low response rate, hence 27% of practices had answered the questionnaire (*N* = 276), 26% of GPs (*N* = 277), and 38% of PNs (*N* = 384) [28]. Four studies were included in the review, three of them originated from UK, and one was from Australia (Fig. 1).

### Analysis and integration of outcomes

The following six themes were identified through the analysis of the results in the included papers: autonomy, professional development, professional status, recognition for work, workload, and Professional identity (see Table 4).

**Table 4:** Findings of the study

First Author/Year	Country	Aim	Method	Sample	Professional tasks	Findings	Themes
Hegney, D. G. 2013 [29]	Australia	The impact of a chronic care management model – nurses’ perceptions and experiences	Semi structured interviews investigating a 12-month intervention of nurse-led care	3 practice managers and 5 nurses	Chronic care Data recording	Nurse-led care influenced job satisfaction positively	
						Opportunity for professional development and autonomy of the practice nurses	Professional development Autonomy
Cousins R. 2012 [13]	UK	To investigate the impact of independent prescribing for experienced nurse practitioners working in general practice.	In-depth interviews	6 nurses	Prescribing of medicine	Prescribing increased levels of job satisfaction among nurses	
						Ability to provide holistic care	Professional development
						Enhanced job control	Autonomy
						Increased status	Professional status
						Increased self-esteem due to patients’ recognition of skills and respect from colleagues	Recognition for work
						Evidence of stressors: lack of reward, increased demands	workload
Maisey, S. 2008 [30]	UK	To understand the effect of payment for performance	Semi structured interviews	1 nurse and 1 GP from 24 practices	Chronic care	Staff:	
						Increased autonomy and responsibility contributed to job satisfaction	Autonomy
						Nurses felt valued as team members	Professional status
						Experienced increased workload	Workload (staff)
						GPs: Only one doctor unequivocally expressed increased job satisfaction Better pay and shorter hours	
						GPs reported a more intensive working pattern as team leaders supervising the nurses’ work.	Workload (GPs)
						Threat to professional identity and values. GPs expressed concerns about loss of continuity and holistic care	Professional identity
McGregor, W. 2008 [31]	UK	To explore views and experiences of role changes under new contract	Semi structured interviews 12–18 months after the Implementa-tion of the new GMS contract Two separate studies, conducted for slightly different aims	18 nurses from different practices (number of practices not informed)	Chronic care Data recording	Practice nurses were positive about their professional role since the introduction of the new GMS contract, which had given them increased responsibility	Autonomy
						Skills enhanced	Professional development
						Their workload and responsibility had increased, but they did not feel rewarded for this, neither financially, nor in terms of involvement in decision making within the practice	Workload Recognition for work

Autonomy in work was a common theme found in all four articles [13, 29–31]. The staff which merely encompassed practice nurses was highly satisfied with their job, and they generally attributed this satisfaction to the autonomy they were granted through delegation of tasks. Autonomy should be understood as the degree to which the staff in general practice are provided freedom, independence, and authority to schedule their own work and make decisions on the care for the patients [32]. The theme “Professional development” was found in three of the included studies [13, 29, 31], and “professional status” was a theme found in two of the studies [13, 30].

In the study by Hegney et al. [29], interviews were conducted with practice managers and nurses pre- and post-trial to explore whether expectations of the implementation of a nurse led model were met. The nurses had been expecting that their new expanded nursing role would improve their work satisfaction as they would become more confident nurses and better communicators. They also believed that it would improve their ability to effectively care for their patients enhancing their skills in chronic disease management for the benefit of the patients receiving a higher standard of care. The interviews with the nurses post-trial showed that their expectations towards their new role had been met.

McGregor et al. [31] explored how nurses in general practice experienced working under the Quality of Outcomes Framework (QOF), which is a pay per performance scheme within the frames of the 2004 General Medical Services contract (GMS) in UK. The QOF incentivise management of certain chronic diseases. The nurses in the study expressed through interviews how their skills and role within their practice had enhanced granting them more autonomy, especially in management of chronic disease. This autonomy was perceived as the main reason for increasing their job satisfaction.

In the study by Cousins et al. [13], being able to finish an episode of care for the patients by writing a prescription was seen by all the nurses to be a main reason for their enhanced job satisfaction. Hence, they perceived this practice to increase their autonomy, their job control, and their ability to provide holistic care to patients. Moreover, the nurses experienced an increase in their status and their self-esteem due to the patients’ recognition of their new extended skills and due to the respect they enjoyed from their colleagues in the clinic.

The nurses in the study by Maisey et al. [30] perceived empowerment of nurses and improvements in teamwork as factors positively influencing the experience of working under the QOF. After implementation of the scheme, the nurses carried out nearly all the routine management of chronic conditions and experienced that it changed the hierarchy within the clinics enhancing their value as team members. They reported that the autonomy they enjoyed in their new positions was closely linked to increased job satisfaction.

The study addressed the views of GPs as well. Even though they agreed with the nurses on the positive influence of task delegation on teamwork and additionally expressed satisfaction with better pay and shorter working hours following the implementation of the QOF, they did not share the overall positive experiences of the new way of working. From the GPs’ perspective the relationship with the patients was influenced negatively as the changes in the working structure challenged the continuity of care. They stated that this loss of continuity prevented them from providing holistic patient care which GPs perceived as a core value. Additionally, they experienced an increase in the workload caused by the delegation of tasks as they had to supervise their staff to a greater extent in providing chronic care for the patients [30]. These findings by Maisey et al. [30] did not appear in any of the other studies.

The nurses had negative experiences and attitudes towards task delegation as well. In three of the studies, the nurses experienced an increase in their workload due to the new working patterns [13, 30, 31]. Even though they generally enjoyed autonomy in their consultations with the patients, some of the nurses in the study by Cousins et al. [13] and the study by McGregor et al. [31] explained that the nurses did not feel rewarded for the extra efforts and responsibilities it imposed on them [13, 31], neither financially nor in terms of advancement [13], nor in involvement in overall organisational decision-making [31].

## Discussion

The nurses were generally satisfied with their job in which they performed various tasks delegated by the GPs. In the nurses’ perspective, this satisfaction was mainly due to the autonomy they enjoyed following the new way of working. However, they experienced an increase in workload and did not feel appreciated for their extra efforts and responsibilities, neither financially, nor in terms of advancement or involvement in decision-making.

Contrary to the nurses, the GPs’ job satisfaction was not increased due to the delegation of tasks to the staff. They experienced a loss of continuity of care threatening their professional identity as providers of holistic care, and they were burdened by an increase in workload due to their new supervisor role.

The results of the included papers regarding the predominant theme, autonomy, are consistent with results of studies conducted among staffs in other clinical settings. Hence, studies across various settings such as the home care environment [33] and different hospital settings [34, 35] have shown that autonomy is one of the most important aspects of the job leading to staff job satisfaction.

Another study explored the experiences and clinical challenges that nurses and nursing assistants face in municipal health service in primary care settings when providing high-quality diabetes care for elderly people [36]. It found that good communication with more experienced health care professionals and access to the right information is particularly important to the staff’s confidence and autonomy in order to make clinical decisions, and that the lack of it was contributory to their experience of not being able to care adequately for the patients. Hence professional development and teamwork were related to the staff’s confidence and experience of autonomy, which they perceived as influencing their provision of high-quality care.

Autonomy being a common theme is in line with existing theory on work motivation. Hackman and Oldham [32] developed a job characteristic model in which certain features of a job could be used as tools for motivating employees as well as for diagnosing existing working conditions. It was tested using data from 658 employees, both blue collar and white collar workers, in seven different business organisations.

The model consisted of five core job dimensions leading to three critical psychological states each contributing to desired personal and work-related outcomes. The five core job dimensions were skill variety, task identity, task significance, autonomy, and feedback. Skill variety, task identity, and task significance together lead to the critical psychological state of meaningfulness of work, autonomy leads to the experienced responsibility for outcomes of the work, and feedback is the only way an employee can obtain knowledge of how well he or she is performing in the job. Each psychological state contributes to high internal work motivation, high quality work performance, high satisfaction with the job, and low absenteeism and turnover [30].

According to the reframing of the model by Dag Ingvar and Jan Thorsvik [37], delegating tasks is a structural feature of an organisation which is characterised by the core job dimension autonomy leading to a critical psychological state of experienced responsibility for outcomes of the work. According to this, task delegation and job satisfaction appear to be interrelated.

In this way, the results of our review seem to support the model by Hackman and Oldham [32], but they do not report on the relative significance of each of the core job dimensions. They solely conclude that the self-generated motivation prompted by the core job dimensions in the model should be highest when all three of the psychological states are present.

However, being a recurring theme in all of the four included papers and in studies conducted in other healthcare settings, autonomy appears to be an essential factor in the job satisfaction of the staff. This relation is not surprising since autonomy allows the individual to influence its own work. Still, autonomy is a subjective phenomenon which differs according to the variation in the individual’s perception of freedom and need for growth in the work [32].

The four included papers varied widely in the methodology and aspects of care, which made it difficult to compare the results. However, the narrative approach used in the review enabled us to explore the views and attitudes of nurses and GPs across these highly different studies even though it did not allow us to standardise the information gained from literature. Yet, by tabulating the evidence in combination with qualitative interpretations of the findings in the form of common themes, we explicated the process from interpretation of the findings towards creation of a synthesis.

A major weakness of the study was that the number of included articles was very small, and therefore, the findings should be interpreted with caution. It is possible that a larger sample would have shown different patterns in the results. Regarding GPs, we only found one paper exploring the relation between task delegation and job satisfaction, hence making it impossible to establish any syntheses on this relation. The language limitation in the study may have influenced the sample size and thereby the studies included. However, since we were interested in international peer-reviewed journals, the vast majority of relevant studies would be in English. We added Scandinavian languages to our search strategy since all of the authors are able to read it and thereby exploring if there were any local studies. However, it did not add any results.

### Interpretation of evidence

Quality of care is essential to the patients and should be taken into consideration when organising general practice. Previous studies have identified medical quality of care and patient satisfaction as important quality parameters, and both have been reviewed in relation to task delegation. Even though job satisfaction has proved to be associated with quality of care, relatively few studies are concerned with this suggested relation.

This review found that task delegation promotes the practice staff’s job satisfaction, and that the reason for this relation first and foremost should be attributed to their experienced autonomy following this new way of working. The study also found that recognition for increased responsibilities should be addressed as well when delegating work to the staff. The validity and credibility of these results were, however, limited by the sample size, and therefore, no final conclusions could be drawn from it.

Moreover, since Hackman and Oldham’s model was tested among employees on various levels in a number of business organisations, it is not necessarily transmissible to the setting of general practice. Therefore, to capture a model which is able to explore the working conditions in general practice, for example how task delegation influences the job satisfaction of GPs and their staff, we need to develop it in this context.

## Conclusions

The studies included in our review suggest that task delegation within general practice may be seen by the staff as an overall positive issue contributing to their job satisfaction, primarily due to perceived autonomy in the work. However, because of the small sample size comprising only qualitative studies, and due to the heterogeneity of these studies, we cannot draw unambiguous conclusions although we point towards tendencies.

The results of the study can be used as the base upon which future studies can build its research with the aim of informing delegation processes within general practice prospectively in order to ensure the staff’s job satisfaction and thereby the quality of care for the patients.

## Abbreviation

GP: General practitioner
